# Supplementary material for: MKP1 promotes nonalcoholic steatohepatitis by suppressing AMPK activity through LKB1 nuclear retention
Source: Nat Commun. 2023 Sep 5;14:5405. doi: 10.1038/s41467-023-41145-5 (PMC10480499; doi:10.1038/s41467-023-41145-5)
Supplement: Supplementary file 1 — Supplementary Information [file 41467_2023_41145_MOESM1_ESM.pdf]

SUPPLEMENTARY MATERIALS

MKP1 promotes nonalcoholic steatohepatitis by suppressing AMPK activity through LKB1 nuclear retention

Bin Qiu[1,2], Ahmed Lawan[3], Chrysovalantou E. Xirouchaki[4,5], Jae-Sung Yi[1,2], Marie Robert[6], Lei Zhang[1,2], Wendy Brown[7], Carlos Fernández-Hernando[2,5,7,8], Xiaoyong Yang[2,8], Tony Tiganis[4,8,9] and Anton M. Bennett[1,2,7,8,*]

[1] Yale University School of Medicine, Department of Pharmacology, 333 Cedar Street, New Haven, CT 06520, U.S.A.

[2] Yale University School of Medicine, Yale Center of Molecular and Systems Metabolism, New Haven, CT 06520, U.S.A.

[3] University of Alabama, Department of Biological Sciences, 301 Sparkman Drive, Huntsville, AL 35899, U.S.A.

[4] Monash Biomedicine Discovery Institute, Monash University, Clayton, Victoria 3800, Australia.

[5] Department of Biochemistry and Molecular Biology, Monash University, Clayton, Victoria 3800, Australia.

[6] Yale University School of Medicine, Department of Pathology, 300 Cedar Street, New Haven, CT 06520, U.S.A.

[7] Monash University Department of Surgery, Alfred Hospital, Melbourne, Victoria, 3004, Australia

[8] Yale University School of Medicine, Vascular Biology and Therapeutics Program, New Haven, CT 06520, U.SA.

[9] Department of Comparative Medicine, Yale University School of Medicine, New Haven, Connecticut, U.S.A.

*To whom correspondence should be addressed.
Anton M. Bennett, Ph.D.
Yale University School of Medicine
Department of Pharmacology
SHM B226D
333 Cedar Street
New Haven, CT 06520-8066
Tel: (203) 737-2441
E-mail: anton.bennett@yale.edu

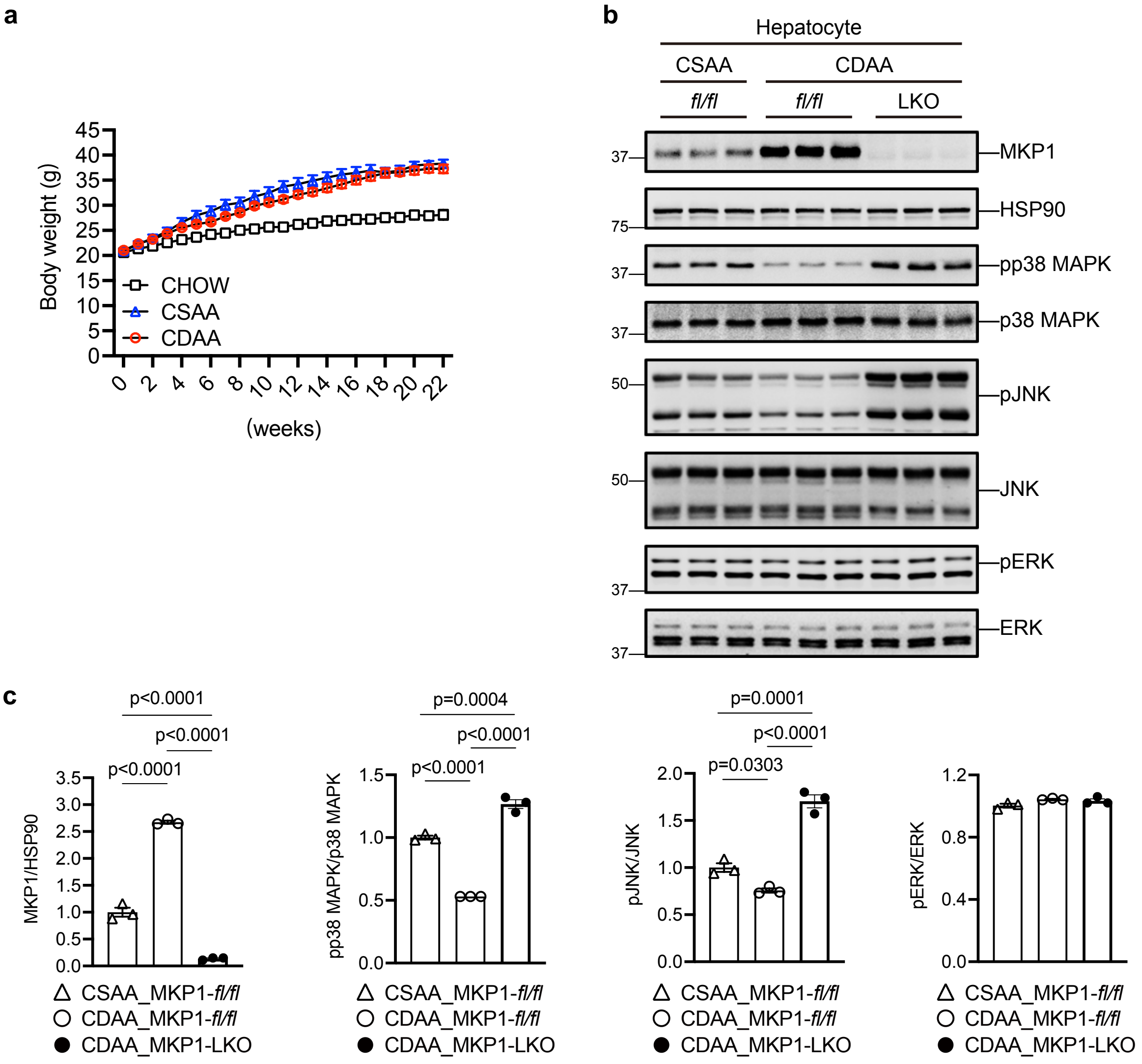

Supplemental Figure 1. MKP1 expression and MAPK regulation in hepatocytes from NASH diet fed mice. (**a**) Growth curve from male *Mkp1*$^{fl/fl}$ mice fed with Chow, CSAA or CDAA diet for 22 weeks. The body weight was recorded weekly. Data represent the mean ± SEM from 10 mice for Chow and CSAA diet, and 13 mice for CDAA diet. (**b**) Immunoblots of MKP1 and HSP90 as a loading control in hepatocytes and p38 MAPK, pJNK and pERK with corresponding MAPK totals from isolated hepatocytes derived from male *Mkp1*$^{fl/fl}$ and MKP1-LKO mice fed with CSAA or CDAA diet for 8 weeks. (**c**) Quantitation of immunoblots from (**b**). Key: *fl/fl, Mkp1*$^{fl/fl}$; LKO, MKP1-LKO. Data represent the mean ± SEM from 3 mice per group. *p* values were determined by one-way ANOVA.

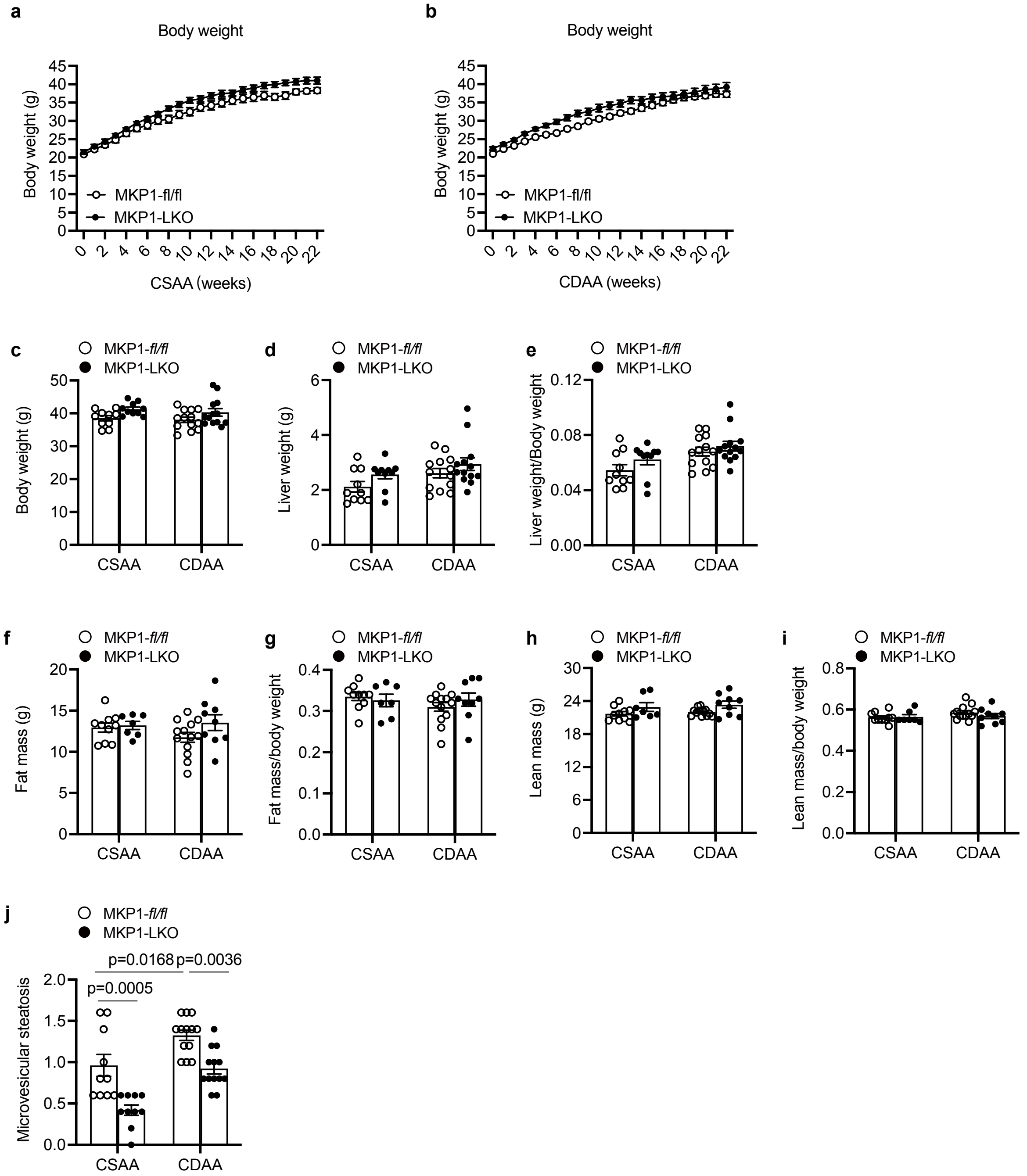

Supplemental Figure 2. Characterization of *Mkp1$^{fl/fl}$* and MKP1-LKO mice fed with CSAA or CDAA diet. (**a-b**) Growth curve from male *Mkp1$^{fl/fl}$* and MKP1-LKO mice fed with CSAA or CDAA diet for 22 weeks, respectively and body weight was recorded weekly. Data in (**a-b**) represent the mean ± SEM from 10 mice for CSAA diet per genotype and 13 mice for CDAA diet per genotype. (**c-e**) Body weight, liver weight and ratio of liver/body weight after 22 weeks of CSSAA and CDAA diet feeding. Data in (**c-e**) represent the mean ± SEM from 10 mice for CSAA diet per genotype and 13 mice for CDAA diet per genotype. *p* values shown were determined by two-way ANOVA. (**f-i**) The body composition in *Mkp1$^{fl/fl}$* and MKP1-LKO mice fed with CSAA or CDAA diet for 22 weeks, presented as (**f**) fat mass, (**g**) fat mass/body weight, (**h**) lean mass and (**i**) lean mass/body weight. Data in (**f-i**) represent the mean ± SEM from 10 mice for CSAA diet-fed *Mkp1$^{fl/fl}$* mice, 7 mice for CSAA diet-fed MKP1-LKO mice, 13 mice for CDAA diet-fed *Mkp1$^{fl/fl}$* mice, 9 mice for CDAA diet-fed MKP1-LKO mice,. (**j**) Quantification of microvesicular steatosis in **Fig. 1c**. Data in (**j**) represent the mean ± SEM from 10 mice for CSAA diet per genotype and 13 mice for CDAA diet per genotype. *p* values shown in (**c**), (**f-j**) were determined by two-way ANOVA, shown in (**d**) and (**e**) were determined by Kruskal-Wallis test.

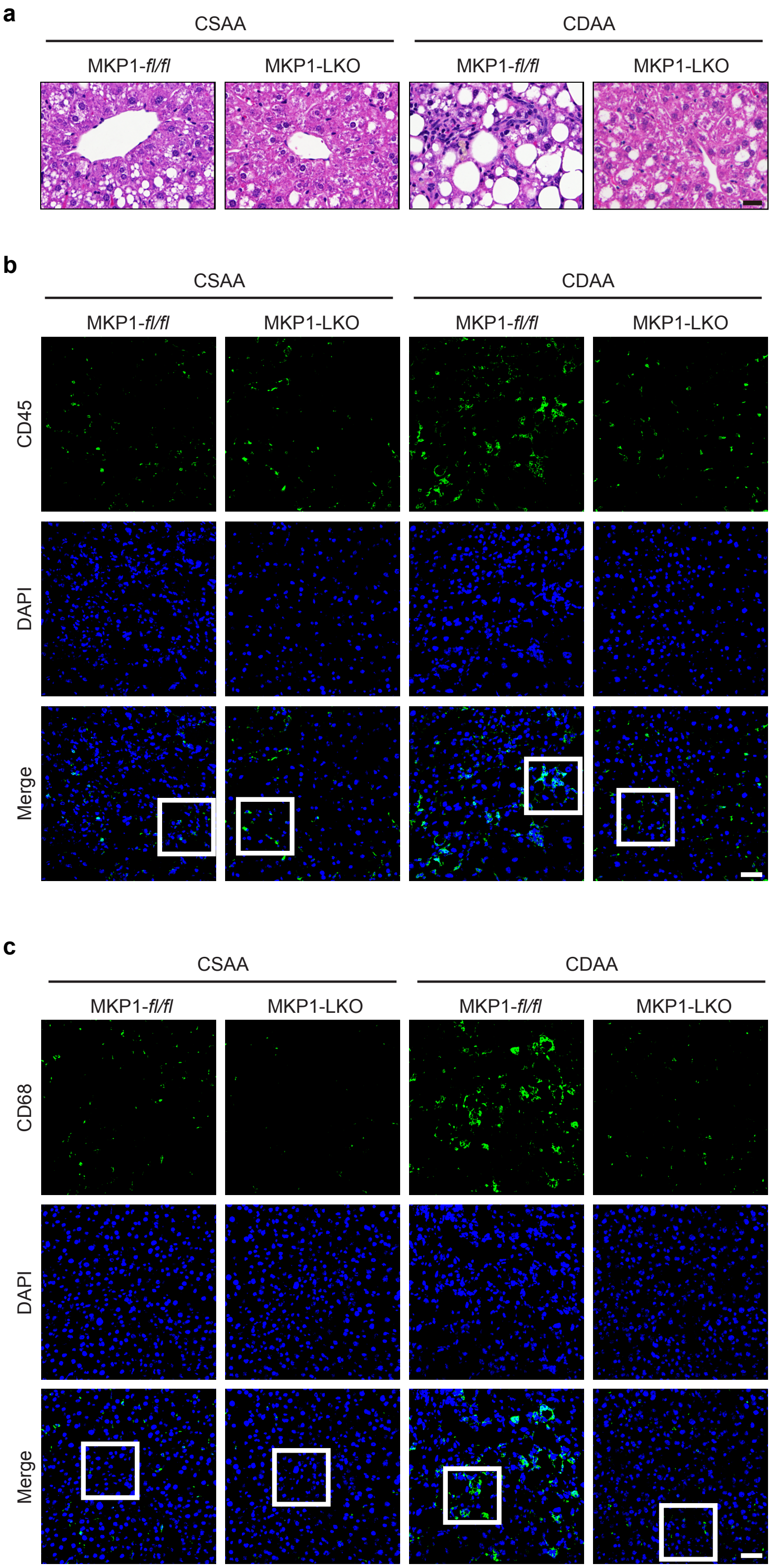

Supplemental Figure 3. Histological analyses of inflammatory infiltrates in MKP1-LKO mice fed with a NASH diet. *Mkp1*$^{fl/fl}$ and MKP1-LKO mice were fed with CSAA or CDAA diet for 22 weeks. (**a**) Histological examination of inflammatory infiltrates in livers. Scale bar = 25 µm. (**b** and **c**) Staining of CD45 or CD68 in liver sections from CSAA or CDAA fed *Mkp1*$^{fl/fl}$ and MKP1-LKO mice. Scale bar = 50 µm. High power magnification of the indicated rectangle areas are shown in **Fig. 3a** and **3d**.

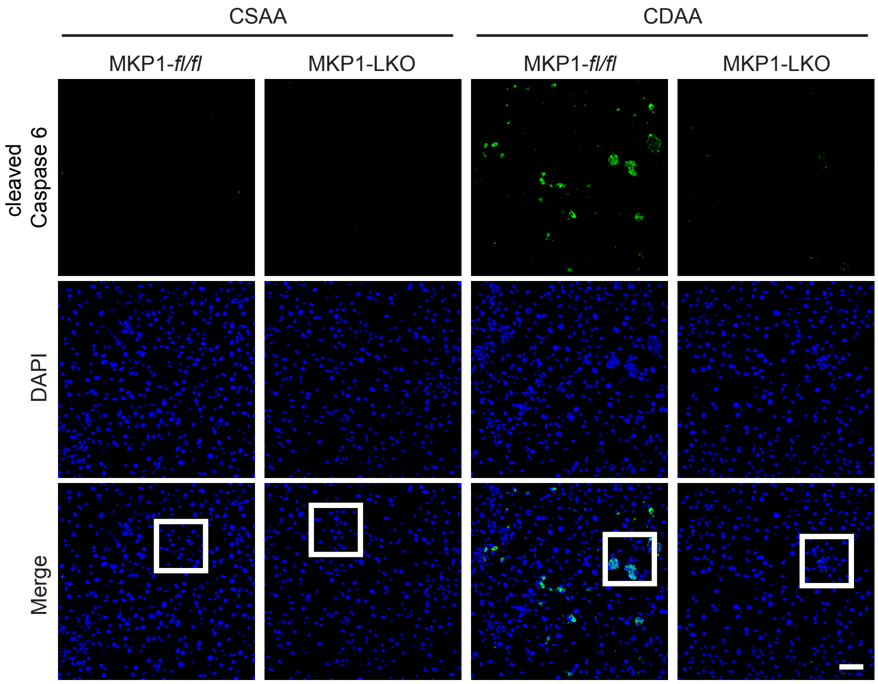

Supplemental Figure 4. MKP1 promotes caspase 6 cleavage in NASH-diet fed mice. Staining of cleaved caspase 6 in liver sections from *Mkp1*$^{fl/fl}$ and MKP1-LKO mice fed with CSAA or CDAA diet for 22 weeks. Scale bar = 50 µm. High power magnification of the indicated rectangle areas are shown in **Fig. 4l**.

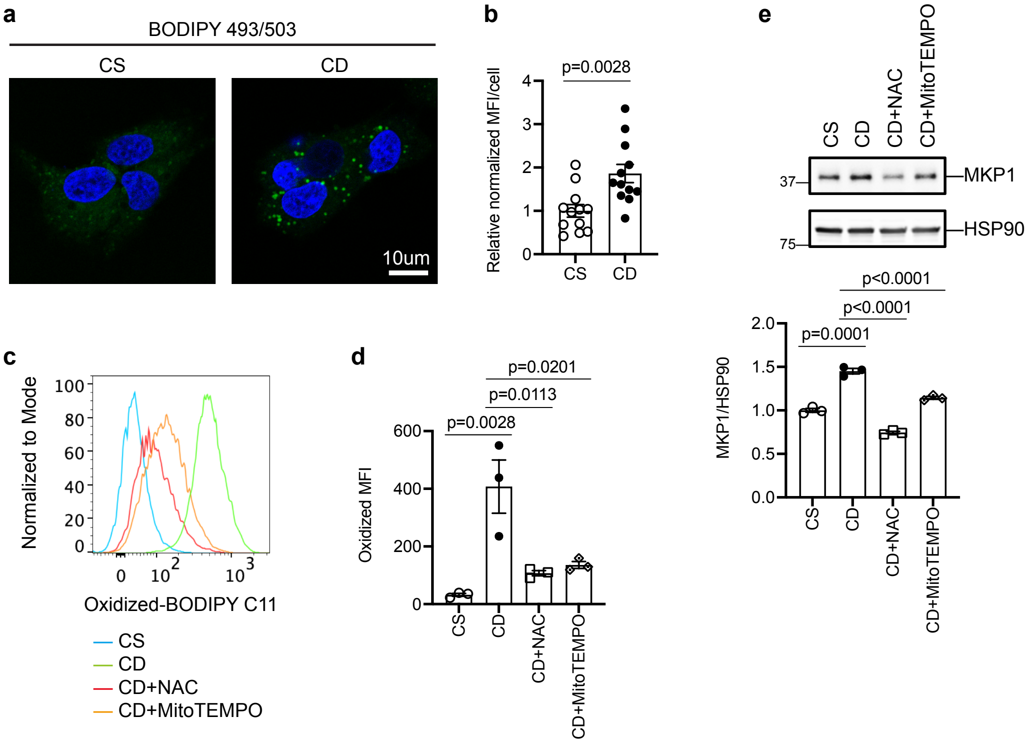

Supplemental Figure 5. MKP1 is upregulated in a ROS-dependent manner in liver cells. HepG2 cells were treated with choline-sufficient (CS) or choline-deficient (CD) medium in the absence or presence of 2 mM NAC or 20 µM MitoTEMPO after 24 h starvation in serum-free medium. (**a**) Lipid content was detected using BODIPY 493/503 (Green) and cell nuclei stained with DAPI (Blue). Scale bar = 10 µm. **(b)** The mean fluorescence intensity (MFI) per field was quantified using ImageJ with each field containing between 2-9 cells. A total of 12 fields were analyzed for each condition (48 cells for CS treated cells and 51 cells for CD treated cells), and MFI values were normalized on a per-cell basis. (**c**) ROS content was measured using BODIPY 581/591 C11. (**d**) MFI signals were calculated by FlowJo. Data represent the mean ± SEM from 3 independent experiments. *p* values were determined by student's-unpaired t test. (**e**) Immunoblots of MKP1 and HSP90 as a loading control. Lower panel represents the quantitation of immunoblots. Data represent the mean ± SEM from 3 independent experiments. *p* values shown in (**b**) were determined by two-sided student's-unpaired t test, shown in (**d**) and (**d**) were determined by one-way ANOVA. Key: CS, choline sufficient medium; CD, choline-deficient medium; NAC, N-acetyl-l-cysteine; MFI, mean fluorescence intensity.

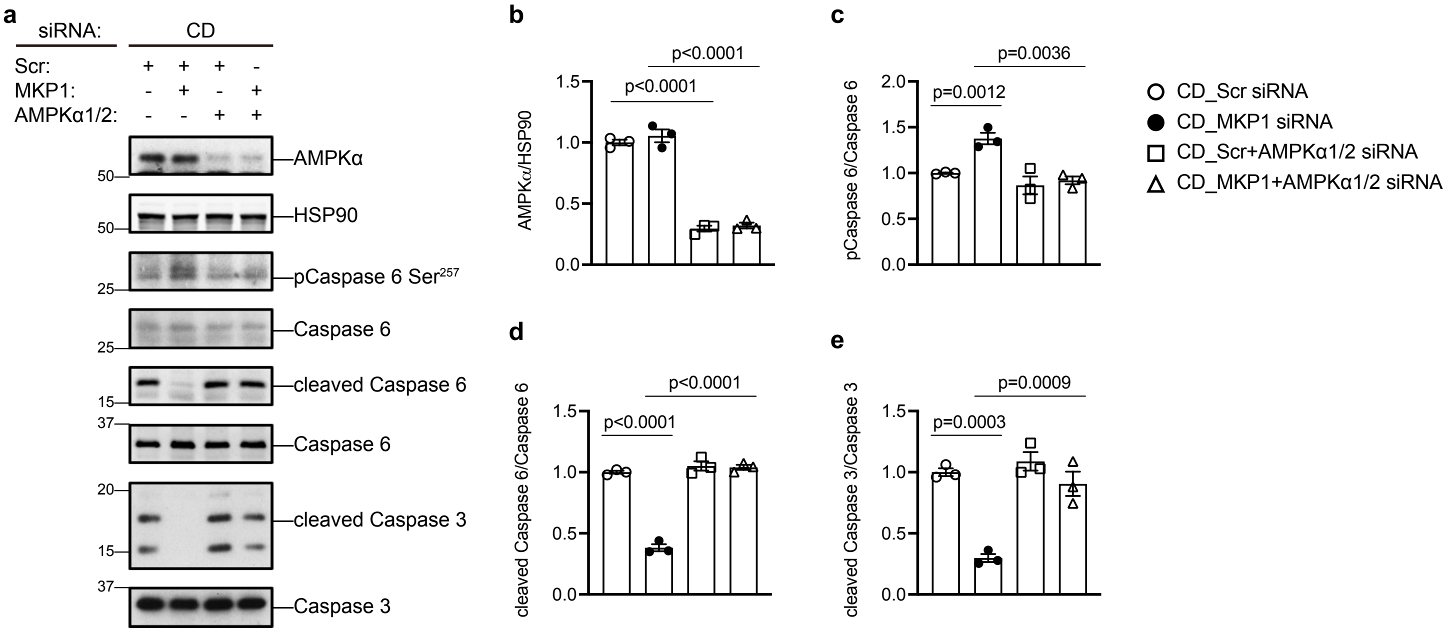

Supplemental Figure 6. MKP1 acts upstream of the AMPKα-caspase 6 pathway. Knockdown of AMPKα1/2 rescues the effects of MKP1 deficiency on Caspase 6/3 cleavage. (**a**) Immunoblots of AMPKα, phospho-caspase 6 (Ser257), cleaved Caspase 6 and cleaved caspase 3 with the indicated corresponding totals. (**b-e**) Quantitation of immunoblots from (**a**). Key: Scr, scrambled siRNA; MKP1, MKP1 siRNA; AMPKα1/2, AMPKα1 siRNA+ AMPKα2 siRNA; CD, choline-deficient medium. Data represent the mean ± SEM from 3 independent experiments. *p* values were determined by two-way ANOVA.

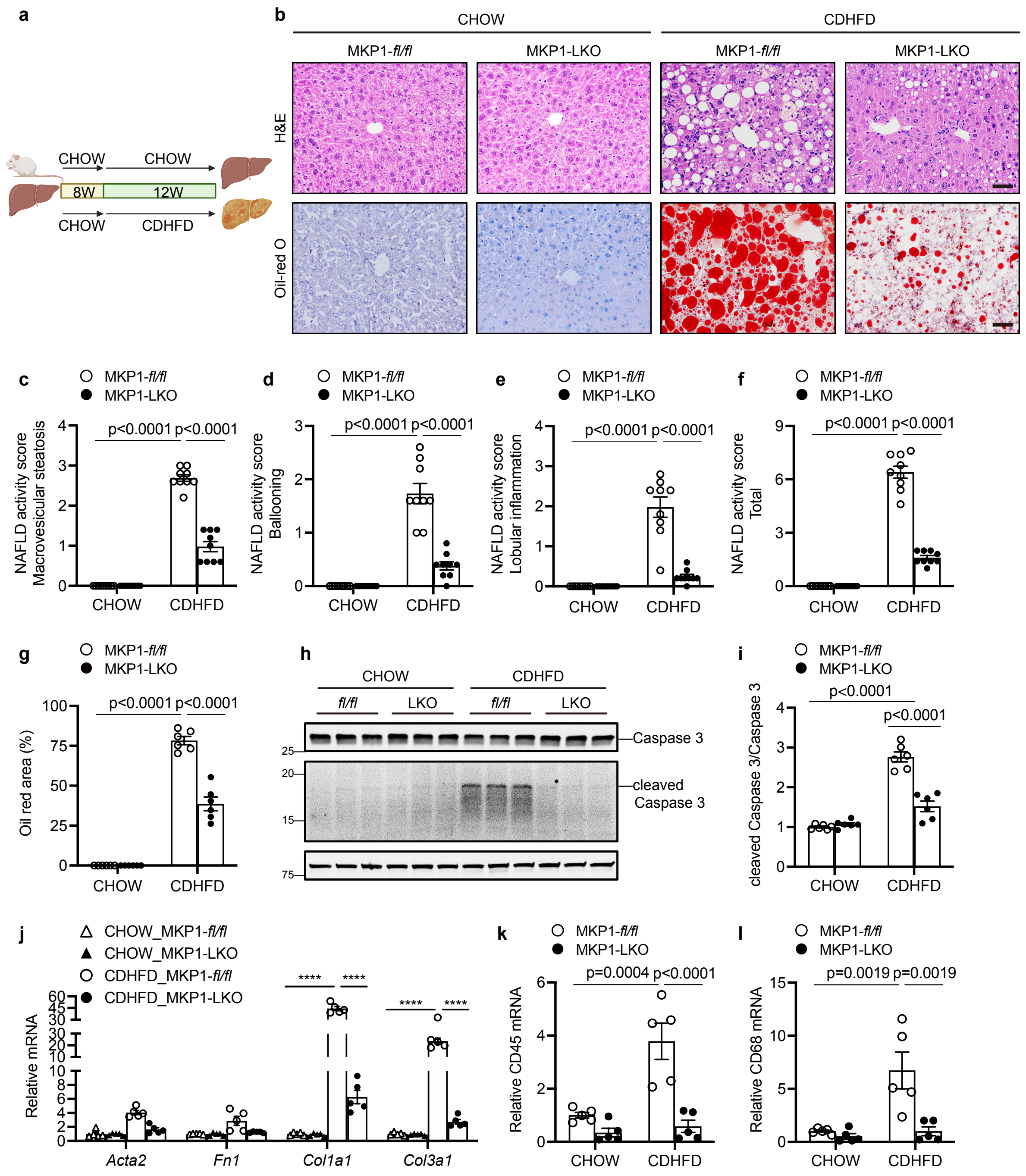

Supplemental Figure 7. Loss of hepatic MKP1 prevents CDHFD diet-induced NASH. The *Mkp1*$^{fl/fl}$ and MKP1-LKO mice were fed with either Chow or CDHFD (60% kcal fat) diet for 12 weeks. (**a**) Schematic diagram for Chow/CDHFD diet. Key: 8W, 8 weeks; 12W, 12 weeks. (**b**) Histological H&E staining (Scale bar = 50 µm) and Oil red-O staining (Scale bar = 50 µm) from liver sections. (**c-f**) NAFLD activity score for macrovesicular steatosis (**c**), ballooning (**d**), lobular inflammation (**e**) and total NAFLD score (**f**) from H&E staining in (**b**). Data in (**c-f**) represent the mean ± SEM derived from 9 mice per genotype. (**g**) Quantification of Oil red O-stained areas from Oil red-O staining in (**b**). Data represent the mean ± SEM derived from 6 mice per group. (**h**) Immunoblot of expression of cleaved caspase 3 and caspase 3 in livers. (**i**) Densitometry of immunoblots from cleaved caspase 3/caspase 3 from (**h**). Data represent the mean ± SEM from 6 mice per group. Key: *fl/fl, Mkp1*$^{fl/fl}$; LKO, MKP1-LKO. (**j**) mRNA expression of fibrotic genes in livers of mice fed chow or CDHFD for 8 weeks. Data represent the mean ± SEM derived from 5 mice per group. (**k and l**) mRNA expression of CD45 and CD68 in livers from Chow or CDHFD fed *Mkp1*$^{fl/fl}$ and MKP1-LKO mice. Data represent the mean ± SEM derived from 5 mice per group. *p* values shown in (**i-l**) were determined by two-way ANOVA, *p* values shown in (**c-f**) were determined by Kruskal-Wallis test.

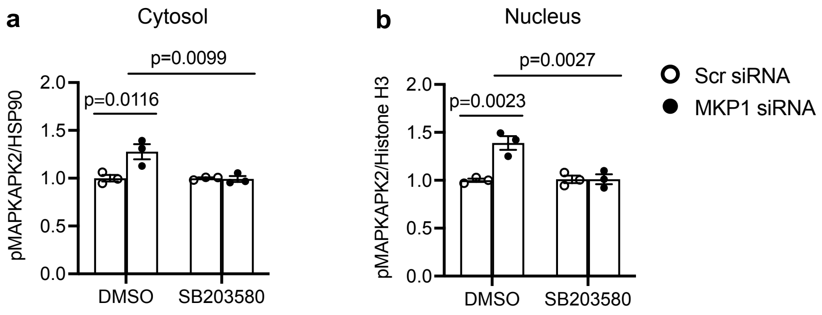

Supplemental Figure 8. MKP1-mediated p38 MAPK activity in SB203580-treated HepG2 cells. Densitometry as a ratio of phospho-MAPKAPK2 (Thr334)/ HSP90 in the cytosol (**a**) and nucleus (**b**) were from immunoblots in **Figure 9a**. Data represent mean ± SEM from 3 independent experiments. *p* values were determined by two-way ANOVA.

Supplemental Figure 9

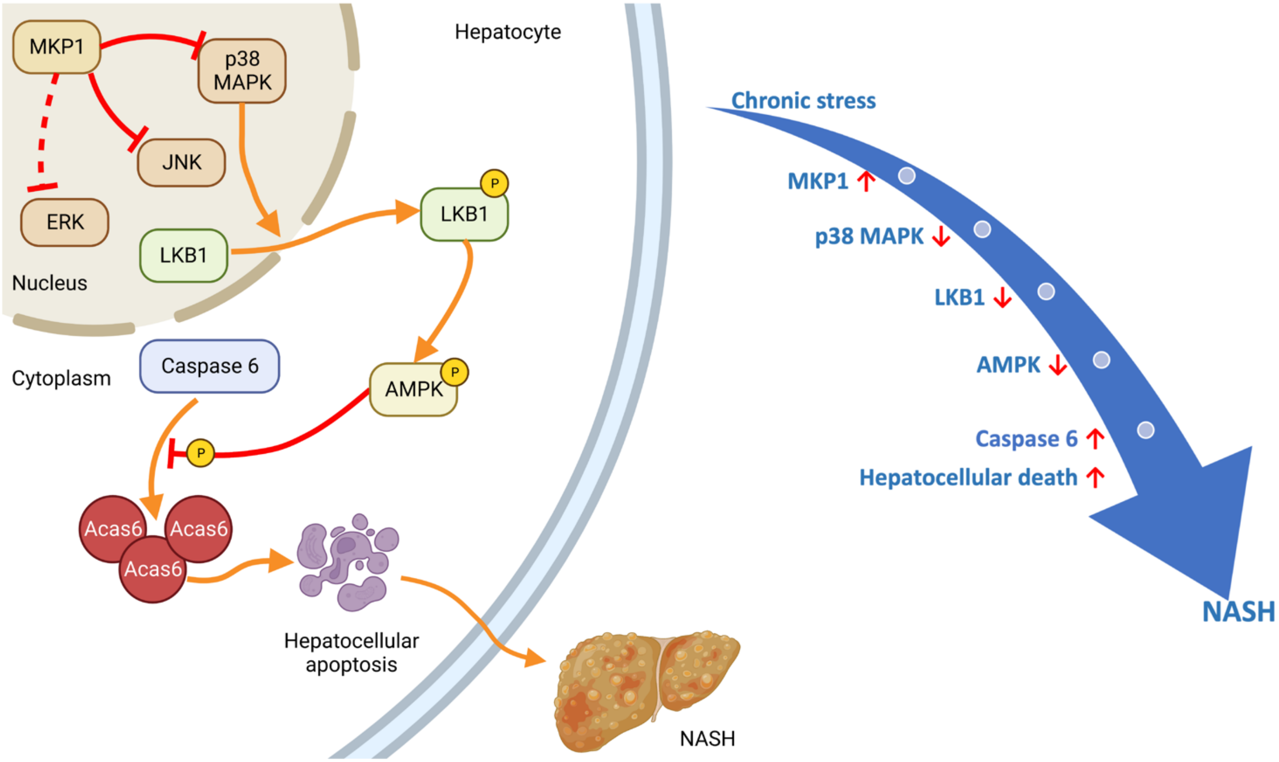

Supplemental Figure 9. Model for the regulation MKP1-p38 MAPK-AMPK-Caspase 6 pathway in NASH. See text for details.

Supplemental Tables

Supplemental Table 1. Primers: primers listed below were from Taqman (Thermo Fisher Scientific)

Gene	Taqman primer
h18S	Cat#Hs99999901_s1
hDUSP1	Cat#Hs00610256_g1
mActa2	Cat#Mm00725412_s1
mCd68	Cat#Mm03047343_m1
mCol1a1	Cat#Mm00801666_g1
mCol3a1	Cat#Mm01254476_m1
mDusp1	Cat#Mm01309843_g1
mFn1	Cat#Mm01256744_m1
mPtprc	Cat#Mm01293577_m1

Supplemental Table 2. Antibodies:

Description	Source	Identifier	WB	IF
AMPKα	Cell Signaling Technology	2603	1:1000	
AMPKβ1/2	Cell Signaling Technology	4150	1:1000	
Anti-mouse IgG, HRP-linked Antibody	Cell Signaling Technology	7076	1:5000	
Anti-rabbit IgG, HRP-linked Antibody	Cell Signaling Technology	7074	1:5000	
Caspase 3	Cell Signaling Technology	9662	1:2000	
Caspase 6	Cell Signaling Technology	9762	1:1000	
CD45	BD Biosciences	ab550539		1:100
CD68	Bio-Rad	MCA1957		1:100
cleaved-caspase 3	Cell Signaling Technology	9664	1:1000	
cleaved-Caspase 6	Cell Signaling Technology	9761	1:500	
cleaved-Caspase 6	GeneTex	GTX59553		1:100

Donkey anti-Rabbit IgG (H+L) Highly Cross-Adsorbed Secondary Antibody, Alexa Fluor 488	Thermo Fisher Scientific	A-21206		1:500
Donkey anti-Sheep IgG (H+L) Cross-Adsorbed Secondary Antibody, Alexa Fluor 594	Thermo Fisher Scientific	A-11016		1:500
ERK1	Santa Cruz Biotechnology	sc94	1:2000	
Flag	Sigma-Aldrich	F3165	1:3000	1:200
Flag-biotin	Sigma-Aldrich	F9219	1:1000	
GAPDH	Santa Cruz Biotechnology	sc137179	1:4000	
HA	Cell Signaling Technology	3724	1:3000	
HA-biotin	Roche	12158167001	1:1000	
Histone-H3	Cell Signaling Technology	4499	1:3000	
HSP90	ProteinTech Group	13171-1-AP	1:4000	
JNK1/2	Santa Cruz Biotechnology	sc571	1:1000	
LKB1	Cell Signaling Technology	3050	1:1000	
LKB1	MRC-PPU Reagents	DA169D		1:100
MKP1	Santa Cruz Biotechnology	sc373841	1:1000	
MKP1	Cell Signaling Technology	48625	1:1000	
p38 MAPK	Santa Cruz Biotechnology	sc81621	1:1000	

phospho-AMPKα (Thr172)	Cell Signaling Technology	2535	1:1000	
phospho-AMPKβ1 (Ser182)	Cell Signaling Technology	4186	1:1000	
phospho-Caspase 6 (Ser 257)	Thermo Fisher Scientific	PA5-12557	1:500	
phospho-ERK1/2 (Thr202/Tyr204)	Cell Signaling Technology	9101	1:1000	
phospho-JNK1/2 (Thr183/Tyr185)	Cell Signaling Technology	4668	1:1000	
phospho-LKB1 (Ser428)	Cell Signaling Technology	3482	1:1000	
phospho-MAPKAPK2 (Thr334)	Cell Signaling Technology	3007	1:1000	
phospho-p38 MAPK (Thr180/Tyr182)	Cell Signaling Technology	4511	1:1000	
phospho-ULK1(Ser555)	Cell Signaling Technology	5869	1:1000	
ULK1	Cell Signaling Technology	8054	1:1000	
α-SMA	Abcam	ab5694	1:2000	
