# Supplementary material for: MKP1 promotes nonalcoholic steatohepatitis by suppressing AMPK activity through LKB1 nuclear retention
Source: Nat Commun. 2023 Sep 5;14:5405. doi: 10.1038/s41467-023-41145-5 (PMC10480499; doi:10.1038/s41467-023-41145-5)
Supplement: Supplementary file 3 — Reporting Summary [file 41467_2023_41145_MOESM3_ESM.pdf]

nature portfolio

Corresponding author(s): Anton M. Bennett

Last updated by author(s): Aug 16, 2023

Reporting Summary

Nature Portfolio wishes to improve the reproducibility of the work that we publish. This form provides structure for consistency and transparency in reporting. For further information on Nature Portfolio policies, see our Editorial Policies and the Editorial Policy Checklist.

Statistics

For all statistical analyses, confirm that the following items are present in the figure legend, table legend, main text, or Methods section.

n/a	Confirmed	
☐	☒	The exact sample size (n) for each experimental group/condition, given as a discrete number and unit of measurement
☐	☒	A statement on whether measurements were taken from distinct samples or whether the same sample was measured repeatedly
☐	☒	The statistical test(s) used AND whether they are one- or two-sided<br>*Only common tests should be described solely by name; describe more complex techniques in the Methods section.*
☒	☐	A description of all covariates tested
☐	☒	A description of any assumptions or corrections, such as tests of normality and adjustment for multiple comparisons
☐	☒	A full description of the statistical parameters including central tendency (e.g. means) or other basic estimates (e.g. regression coefficient) AND variation (e.g. standard deviation) or associated estimates of uncertainty (e.g. confidence intervals)
☐	☒	For null hypothesis testing, the test statistic (e.g. F, t, r) with confidence intervals, effect sizes, degrees of freedom and P value noted<br>*Give P values as exact values whenever suitable.*
☒	☐	For Bayesian analysis, information on the choice of priors and Markov chain Monte Carlo settings
☒	☐	For hierarchical and complex designs, identification of the appropriate level for tests and full reporting of outcomes
☒	☐	Estimates of effect sizes (e.g. Cohen's d, Pearson's r), indicating how they were calculated

Our web collection on statistics for biologists contains articles on many of the points above.

Software and code

Policy information about availability of computer code

Data collection	No custom code used in this manuscript.
Data analysis	GraphPad Prism 9 Version 9.5.1 (GraphPad Software Inc.)

For manuscripts utilizing custom algorithms or software that are central to the research but not yet described in published literature, software must be made available to editors and reviewers. We strongly encourage code deposition in a community repository (e.g. GitHub). See the Nature Portfolio guidelines for submitting code & software for further information.

Data

Policy information about availability of data

All manuscripts must include a data availability statement. This statement should provide the following information, where applicable:
- Accession codes, unique identifiers, or web links for publicly available datasets
- A description of any restrictions on data availability
- For clinical datasets or third party data, please ensure that the statement adheres to our policy

All data needed to evaluate the conclusions in the paper are presented and additional information ca

Research involving human participants, their data, or biological material

Policy information about studies with human participants or human data. See also policy information about sex, gender (identity/presentation), and sexual orientation and race, ethnicity and racism.

Reporting on sex and gender	Depending on availability, liver biopsies from either male or female subjects were processed and used in experiments. Due to the low frequency of suitable donors analyses on gender differences were not performed.
Reporting on race, ethnicity, or other socially relevant groupings	Due to the low frequency of suitable donors analyses on race, ethnicity and other grouping were not performed.
Population characteristics	13 females, 8 males, BMIs 36-74
Recruitment	Liver core biopsies from obese men and women undergoing bariatric surgery taken on the day of surgery.
Ethics oversight	The use of human tissue was approved by the Monash University Human Research Ethics Committee (CF12/2339-2012001246; CF15/3041-2015001282).

Note that full information on the approval of the study protocol must also be provided in the manuscript.

Field-specific reporting

Please select the one below that is the best fit for your research. If you are not sure, read the appropriate sections before making your selection.

- [x] Life sciences
- [ ] Behavioural & social sciences
- [ ] Ecological, evolutionary & environmental sciences

For a reference copy of the document with all sections, see nature.com/documents/nr-reporting-summary-flat.pdf

Life sciences study design

All studies must disclose on these points even when the disclosure is negative.

Sample size	Sample size was not pre-determined. Sample size used was based upon previous experiments in which the defined sample size was sufficient to power statistical significance.
Data exclusions	None.
Replication	All experiments in vitro and in cell based models were performed at least three time independently. In vivo mouse models used at least 5 mice per genotype. All experimental repetitions were successfully performed.
Randomization	All data were first subjected to a normality test. For parametric data, the differences between two groups were compared by unpaired student's t-test, or among multiple groups were analyzed by one-way or two-way analysis of variance (ANOVA) combined with Tukey's post-hoc test. For nonparametric data, the differences between two groups were compared by Mann-Whitney test, or among multiple groups were analyzed by Kruskal-Wallis test. The $p \leq 0.05$ was considered statistically significant.
Blinding	Blinding in cell culture and animal model experiments was not practically feasible. Further data output was quantitative and not subject to bias.

Reporting for specific materials, systems and methods

We require information from authors about some types of materials, experimental systems and methods used in many studies. Here, indicate whether each material, system or method listed is relevant to your study. If you are not sure if a list item applies to your research, read the appropriate section before selecting a response.

Materials & experimental systems

n/a	Involved in the study	
☐	☒	Antibodies
☐	☒	Eukaryotic cell lines
☒	☐	Palaeontology and archaeology
☐	☒	Animals and other organisms
☒	☐	Clinical data
☒	☐	Dual use research of concern
☒	☐	Plants

Methods

n/a	Involved in the study	
☒	☐	ChIP-seq
☐	☒	Flow cytometry
☒	☐	MRI-based neuroimaging

Antibodies

Antibodies used	AMPKα Cell Signaling Technology 2603 1:1000<br>AMPKβ1/2 Cell Signaling Technology 4150 1:1000<br>Anti-mouse IgG, HRP-linked Antibody Cell Signaling Technology 7076 1:5000<br>Anti-rabbit IgG, HRP-linked Antibody Cell Signaling Technology 7074 1:5000<br>Caspase 3 Cell Signaling Technology 9662 1:2000<br>Caspase 6 Cell Signaling Technology 9762 1:1000<br>CD45 BD Biosciences ab550539 1:100<br>CD68 Bio-Rad MCA1957 1:100<br>cleaved-caspase 3 Cell Signaling Technology 9664 1:1000<br>cleaved-Caspase 6 Cell Signaling Technology 9761 1:500<br>cleaved-Caspase 6 GeneTex GTX59553 1:100<br>Donkey anti-Rabbit IgG (H+L) Highly Cross-Adsorbed Secondary Antibody, Alexa Fluor 488 Thermo Fisher Scientific A-21206 1:500<br>Donkey anti-Sheep IgG (H+L) Cross-Adsorbed Secondary Antibody, Alexa Fluor 594 Thermo Fisher Scientific A-11016 1:500<br>ERK1 Santa Cruz Biotechnology sc94 1:2000<br>Flag Sigma-Aldrich F3165 1:3000 1:200<br>Flag-biotin Sigma-Aldrich F9219 1:1000<br>GAPDH Santa Cruz Biotechnology sc137179 1:4000<br>HA Cell Signaling Technology 3724 1:3000<br>HA-biotin Roche 12158167001 1:1000<br>Histone-H3 Cell Signaling Technology 4499 1:3000<br>HSP90 ProteinTech Group 13171-1-AP 1:4000<br>JNK1/2 Santa Cruz Biotechnology sc571 1:1000<br>LKB1 Cell Signaling Technology 3050 1:1000<br>LKB1 MRC-PPU Reagents DA169D 1:100<br>MKP1 Santa Cruz Biotechnology sc373841 1:1000<br>MKP1 Cell Signaling Technology 48625 1:1000<br>p38 MAPK Santa Cruz Biotechnology sc81621 1:1000<br>phospho-AMPKα (Thr172) Cell Signaling Technology 2535 1:1000<br>phospho-AMPKβ1 (Ser182) Cell Signaling Technology 4186 1:1000<br>phospho-Caspase 6 (Ser 257) Thermo Fisher Scientific PA5-12557 1:500<br>phospho-ERK1/2 (Thr202/Tyr204) Cell Signaling Technology 9101 1:1000<br>phospho-JNK1/2 (Thr183/Tyr185) Cell Signaling Technology 4668 1:1000<br>phospho-LKB1 (Ser428) Cell Signaling Technology 3482 1:1000<br>phospho-MAPKAPK2 (Thr334) Cell Signaling Technology 3007 1:1000<br>phospho-p38 MAPK (Thr180/Tyr182) Cell Signaling Technology 4511 1:1000<br>phospho-ULK1(Ser555) Cell Signaling Technology 5869 1:1000<br>ULK1 Cell Signaling Technology 8054 1:1000<br>α-SMA Abcam ab5694 1:2000
Validation	Cell Signaling Technology provide a validation procedure and statement (https://www.cellsignal.com/about-us/cst-antibody-validation-principles?_requestid=1590519). Antibody validations can also be found at the companies website in the data sheet for each antibody online (https://www.cellsignal.com/).<br>Santa Cruz Biotechnology antibody validations can be found at the companies website in the data sheet for each antibody (https://www.scbt.com/home).<br>BD Biosciences antibody validations can be found at the companies website in the data sheet for each antibody (https://www.bdbiosciences.com/en-us).<br>Bio-Rad provide a validation procedure and statement (https://www.bio-rad-antibodies.com/our-antibody-validation-principles.html). Antibody validations can also be found at the companies website in the data sheet for each antibody online (https://www.bio-rad-antibodies.com/primary-antibodies-monoclonal-polyclonal.html).<br>GeneTex antibody validations can be found at the companies website in the data sheet for each antibody (https://www.genetex.com/).<br>Thermo Fisher Scientific validations can be found at the companies website in the data sheet for each antibody (https://www.thermofisher.com/us/en/home.html).<br>Sigma-Aldrich provide a validation statement (https://www.sigmaaldrich.com/US/en/products/protein-biology/antibodies/enhanced-validation-ab). Antibody validations can also be found at the companies website in the data sheet for each antibody online (https://www.sigmaaldrich.com/US/en/products).<br>Roche validations can be found at the companies website in the data sheet for each antibody (https://diagnostics.roche.com/).<br>MRC-PPU Reagents validation (no information provided)<br>Abcam provide a validation procedure and statement (https://www.abcam.com/primary-antibodies/a-guide-to-antibody-validation). Antibody validations can also be found at the companies website in the data sheet for each antibody online (https://www.abcam.com/).

Eukaryotic cell lines

Policy information about cell lines and Sex and Gender in Research

Cell line source(s)	HepG2 cell line (# HB-8065; ATCC)
Authentication	As per ATCC authentication description (https://www.atcc.org/products/hb-8065)<br>STR profiling<br>Amelogenin: X,Y<br>CSF1PO: 10,11<br>D13S317: 9,13<br>D16S539: 12,13<br>D5S818: 11,12<br>D7S820: 10<br>TH01: 9<br>TPOX: 8,9<br>vWA: 17<br>D3S1358: 15,16<br>D21S11: 29,31<br>D18S51: 13,14<br>Penta_E: 15,20<br>Penta_D: 9,13<br>D8S1179: 15,16<br>FGA: 22,25<br>D19S433: 15.2<br>D2S1338: 19,20
Mycoplasma contamination	Negative mycoplasma contamination.
Commonly misidentified lines<br>(See ICLAC register)	None

Animals and other research organisms

Policy information about studies involving animals; ARRIVE guidelines recommended for reporting animal research, and Sex and Gender in Research

Laboratory animals	Male Mkp1fl/fl and MKP1-LKO mice
Wild animals	No wild animals were used in these studies.
Reporting on sex	Only male mice were used in the study.
Field-collected samples	No field collected samples were used in this study.
Ethics oversight	Yale University School of Medicine Institutional Animal Care and Use Committee approved all animal studies.

Note that full information on the approval of the study protocol must also be provided in the manuscript.

Flow Cytometry

Plots

Confirm that:

- [x] The axis labels state the marker and fluorochrome used (e.g. CD4-FITC).
- [x] The axis scales are clearly visible. Include numbers along axes only for bottom left plot of group (a 'group' is an analysis of identical markers).
- [x] All plots are contour plots with outliers or pseudocolor plots.
- [x] A numerical value for number of cells or percentage (with statistics) is provided.

Methodology

Sample preparation	HepG2 cells were subjected to the specified treatments. Subsequently, the cells were incubated with BODIPY™ 581/591 C11 at a final concentration of 5 μM. The incubation was carried out for 30 minutes at 37 ℃ in the absence of light. Afterward, the cells were trypsinized and gathered for analysis using FACS. Unstained cells were used for negative control and gating.
Instrument	LSRII, BD Biosciences

Software	FlowJo 10.9.0.
Cell population abundance	The experiment was exclusively conducted using pure HepG2 cell populations.
Gating strategy	1) Identification of living cells was accomplished based on FSC-A (Forward Scatter-A)/SSC-A (Side Scatter-A) characteristics. 2) Subsequently, single cells were identified using FSC-A/FSC-H (Forward Scatter-A/Forward Scatter-H) parameters. 3) Detection of cells exhibiting oxidized fluorescence was achieved through the use of BODIPY™ 581/591 C11, with excitation at 488 nm and emission at 510 nm. 4) The demarcation between "positive" and "negative" cells was established by considering the signal exceeding that of the negative cell population.

☐ Tick this box to confirm that a figure exemplifying the gating strategy is provided in the Supplementary Information.
